# Habitat Size and Location Drive Heterogeneity in Oyster Shell Colonization by Sessile Invertebrates

**DOI:** 10.1002/ece3.71683

**Published:** 2025-07-09

**Authors:** Elizabeth A. Hamman

**Affiliations:** ^1^ Department of Biology St. Mary's College of Maryland St. Mary's City Maryland USA

**Keywords:** colonization, oyster reef, patch selection, patch size, sessile

## Abstract

Oyster reefs form a critical, biogenic coastal habitat and host diverse assemblages of fish and invertebrates. Previous studies show that variation in the settlement and distribution of oyster reef inhabitants depends on factors such as flow and members of the benthic community. In other reef systems, such as coral reefs, the proximity of neighboring reefs also affects these patterns, yet this phenomenon is less explored in oyster systems. In this study, we tested the effects of habitat size and location on the colonization of sessile organisms living on restored oyster reef habitat. We placed cages filled with oyster shells of two sizes at two distances from the restored reef on the waterfront of St. Mary's College of Maryland. After 3 months, we collected the cages and identified and counted the individuals that colonized the shell. We found organisms responded differently to habitat size and location. For example, hooked mussel ( *Ischadium recurvum* ) abundance was primarily driven by proximity to the restored reef rather than the amount of available habitat. In contrast, *Balanus* spp. abundance was affected mainly by habitat size rather than location. Community composition depended only on habitat size. Therefore, habitat size and location can play an essential role in the community assembly of added oyster habitats through their taxon‐specific effects.

## 1 Introduction

Many marine habitats are heterogeneous in space and are composed of patches that vary in size and isolation. Along with this heterogeneity in habitat across the seascape, organisms vary in abundance across habitat patches, and communities differ in diversity. Heterogeneity in the habitat often drives the heterogeneity of the organisms that occupy it, as often seen in studies focusing on habitat fragmentation. For example, a meta‐analysis of fragmentation in marine habitats found landscapes of smaller, isolated habitats often, but not always, have less diverse communities or host fewer organisms than landscapes with the same habitat area but arranged in larger, less isolated patches (Yeager et al. 2020). At the population level, responses to habitat configuration are highly variable across taxa (Eggleston 1999; Harwell et al. 2011; Yarnall et al. 2022), leading to various spatial distributions across marine organisms.

Oyster reefs are frequently fragmented (Benson et al. 2023), heterogeneous habitats composed of individual habitat patches. As a result, metapopulation theory and insights from landscape ecology are increasingly used in their restoration (Eggleston 1999; Schulte et al. 2009). Both patch size and context within the landscape affect oyster recruitment and survival and, therefore, restoration success (Caretti et al. 2024). Oyster restoration efforts aim not only to restore the oysters themselves but also the communities they support (Zu Ermgassen et al. 2016). Oyster reefs host diverse assemblages of fish and invertebrates that vary in space (Grabowski et al. 2020; Gregalis et al. 2009; Shervette and Gelwick 2008). This variation is often attributed to variation in habitat patch characteristics and context within the landscape, but the degree of this depends on the focal organism. For example, neighboring seagrass meadows can increase the abundance of certain fish, but not the overall abundance or community composition in the Noosa River of eastern Australia (Gilby et al. 2019). Yet, in another location, landscape context (neighboring mudflat vs. seagrass/salt marsh) explained oyster populations and invertebrate occupant communities but not fish communities (Ziegler et al. 2018).

The variation in an organism's spatial distribution across a patchy landscape depends on factors that affect either the organism's colonization of a patch or its survival in the patch post‐colonization. For example, blue crab megalopae avoid the scent of one of their predators. As a result, they are less likely to colonize patches with the predator present (Welch et al. 1997), generating heterogeneity among patches in the landscape. Additionally, variation in post‐colonization processes, such as growth or survival, can generate heterogeneity. Blue crab survival varies across patch size and season (Hovel and Lipcius 2001), generating spatial variability in blue crab density across the landscape. While both colonization and post‐colonization play a key role in the spatial distribution of blue crabs, most variation is due to differences in patch colonization (Moksnes and Heck 2006).

The effects of habitat characteristics on the settlement or colonization of a focal patch are significant for sessile organisms, who cannot move to a better patch if they make a poor selection. The settlement patterns of sessile organisms on oyster reefs depend on factors such as chemical signals (e.g., pheromones from barnacle conspecifics, Dreanno et al. 2006a, 2006b) and the presence of specific community members such as predators (Pruett and Weissburg 2019). Additionally, abiotic factors such as substrate type and microhabitat conditions such as light and sedimentation (Michener and Kenny 1991) can affect settlement patterns.

The effects of habitat size and location of a habitat patch within the landscape can also be important for colonization. For example, barnacles have lower densities on habitat patches located further from the larval source than those near the source. This difference occurs because “settlement shadows” are formed, and larval densities are depleted as the water moves over habitat patches (Gaines et al. 1985). Therefore, a habitat patch that is isolated from a larger habitat patch that is the source of dispersers would likely receive fewer colonists. Habitat size plays an important role as well. Larger habitats might be more attractive overall and can have higher densities of organisms than smaller habitats, indicating an appeal of a larger habitat beyond simply an increase in area (Eggleston et al. 1999; Hanke et al. 2017). Habitat size and location are also likely to interact to influence community composition. Habitats with higher larval input might be more likely to “fill up” with settlers, potentially favoring those that arrive earlier in the season. The size of a habitat patch would likely influence the strength of interactions post‐colonization as the organisms compete for space and food. While both habitat size and location have demonstrated effects on the colonization of specific organisms that inhabit oyster reefs, their combined effects across the entire community of sessile fauna on an oyster reef are unknown.

Multiple hypotheses predict different outcomes in the number of individuals per patch due to variation in habitat size and location among otherwise identical patches. There are explanations for the number of colonists to increase or decrease as habitat size and location change. For example, the field of dreams hypothesis predicts that increasing habitat size will proportionately increase the number of occupants (Palmer et al. 1997). Therefore, the location of a habitat patch in a landscape will not affect the number of colonists it receives, as each habitat receives an amount proportional to its area. This outcome contrasts with that expected under the propagule redirection hypothesis. Under propagule redirection, colonists that arrive in a given area are shared among the available habitats. Therefore, the number of settlers is fixed in a region regardless of whether there is one large or two small habitats near one another (Stier and Osenberg 2010). Other theories are specific to the position of a patch relative to the larval source. Habitats that are isolated from source populations are often less likely to be colonized (Gaines et al. 1985; MacArthur and Wilson 1967) but could also benefit from having less competition for available settlers under propagule redirection and receive more settlers (Stier and Osenberg 2010). Thus, depending on larval behavior, settlement cues, and survival in the immediate aftermath of settlement, small habitats further from the restored reef and larval source might host the fewest or most settlers.

This study examines the effects of habitat size and location across the sessile fauna community near a restored oyster reef. We test whether habitat size and proximity to a restored oyster reef affects (1) community composition and diversity among the sessile faunal community and (2) taxon‐specific spatial distributions.

## 2 Methods

### 2.1 Field Experiment

**FIGURE 1:**
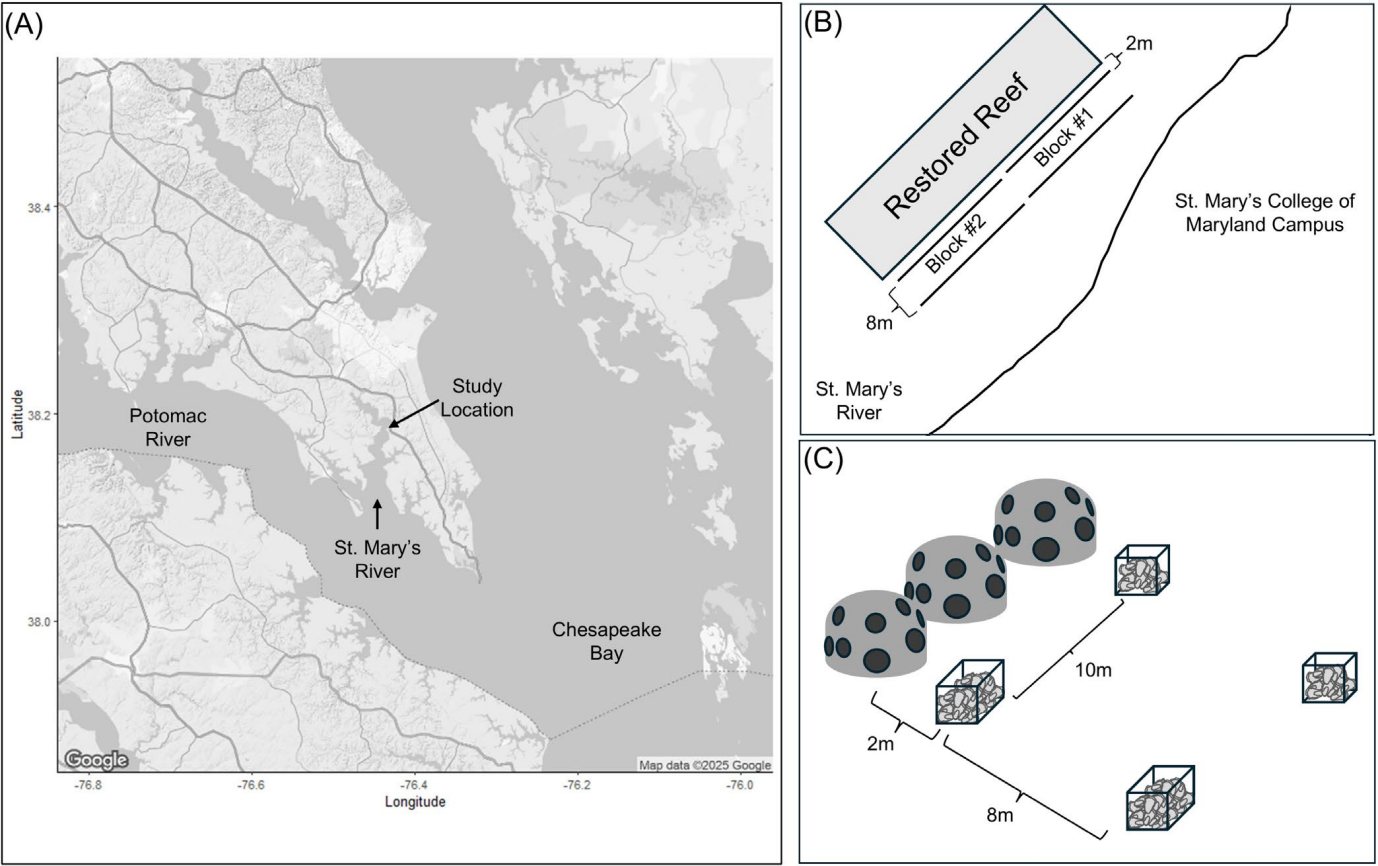
Experimental location and design. (A) Indicates the study location in St. Mary's River, and (B) shows the experimental layout relative to the restored reef and shoreline. The cages of oyster shell were deployed in two lines (“near” and “far” from the restored reef) and in two spatial and temporal blocks. (C) Cages in the “far” location were placed directly behind cages of the same size.

This study took place in the St. Mary's River (38°11′31″N 76°25′50″W) at depths ranging from 2 to 3 m (Figure 1A) near a restored reef constructed of added shell, reef balls, and concrete rubble. To test the effects of habitat size and location on sessile colonizers of oyster shells, we deployed cages constructed of 1‐inch vinyl‐coated galvanized steel mesh and filled with aged oyster shell. Large cages measured 20.25 cm by 20.25 cm by 40.5 cm and contained 40 shells, while small cages measured 20.25 cm by 20.25 cm by 20.25 cm and contained 20 shells. A doubling of habitat amount is a ratio previously used in studies of added oyster habitat (Kimbro et al. 2020). Before adding the shell to the cages, we sorted the shell into size classes, which were evenly distributed among the cages to minimize differences in settlement due to variations in surface area.

We deployed the cages in two parallel lines (Figure 1B) between the restored reef and the shore so that both sources (the restored reef and the river) were in the same direction of the experiment. “Near” cages were placed approximately 2 m from the reef to ensure no cages were placed on top of added reef materials, and “far” cages approximately 10 m from the reef. Previous studies of neighboring habitats on colonization in marine environments have ranged from effects of neighboring habitats at 2 m, and assumed no effect at 16 m (Stier and Osenberg 2010), assumed effect at 5 m and none at 20 m (Morton and Shima 2013). We chose our distance of 10 m to fall within these ranges and while maintaining consistent depth between each pair of near and far cages to reduce the effect of shallower water nearer to shore affecting our results. We placed each “far” cage behind a cage of the same size (i.e., the order of cage size was the same for both the near and far cage lines, Figure 1C). The experiment was deployed and collected in two spatial and temporal blocks (Figure 1B) to account for variations in hydrological features and the restored reef at the site and any differences in colonization between the two deployment and collection dates (separated by 1 week). After 3 months of colonization during the summer months, we returned the cages to shore in individual containers. All shells were rinsed, and the organisms settled on the shell were identified and counted. We excluded three cages from the final analysis due to loss (1 cage) or burial in the sediment (2 cages).

### 2.2 Statistical Analysis

We used generalized linear models to test the effects of habitat size and location on three diversity metrics and populations of the sessile colonizers of oyster shell. Our model included habitat size and location as predictor variables and their interaction. Because we deployed the experiment in two spatial and temporal blocks, we included block ID in our models, which had two levels with four replicates each. Because block ID only had two levels, we included it in our models as a fixed effect, rather than a random effect. We calculated the overall abundance, species richness, and Shannon's H as response variables for each cage to examine the effects of habitat size and location on the sessile community. We assumed a Gaussian distribution for overall abundance and Shannon's H, and a quasi‐Poisson distribution for richness due to under‐dispersed count. To look at species‐specific responses, we performed the same analysis based on the counts of each species. All counts were over‐dispersed, so we assumed a negative binomial distribution, and oyster and serpulid counts required the inclusion of a zero‐inflation term. Depending on the required distribution, we used either the MASS (e.g., quasi‐Poisson) or glmmTMB packages (e.g., zero‐inflated Negative Binomial) (Brooks et al. 2024; Ripley and Venables 2009). We used the DHARMa package (Hartig and Lohse 2022) to validate all generalized linear models. To test the effects of habitat size and location on community composition, we used a PERMANOVA with both the main and interactive effects of size and location along with block ID implemented in the vegan package with Bray‐Curtis distances (Oksanen et al. 2001) and visualized the communities using nMDS. We conducted all analyses in R Version 4.4.1 (R Core Team 2024).

## 3 Results

**FIGURE 2:**
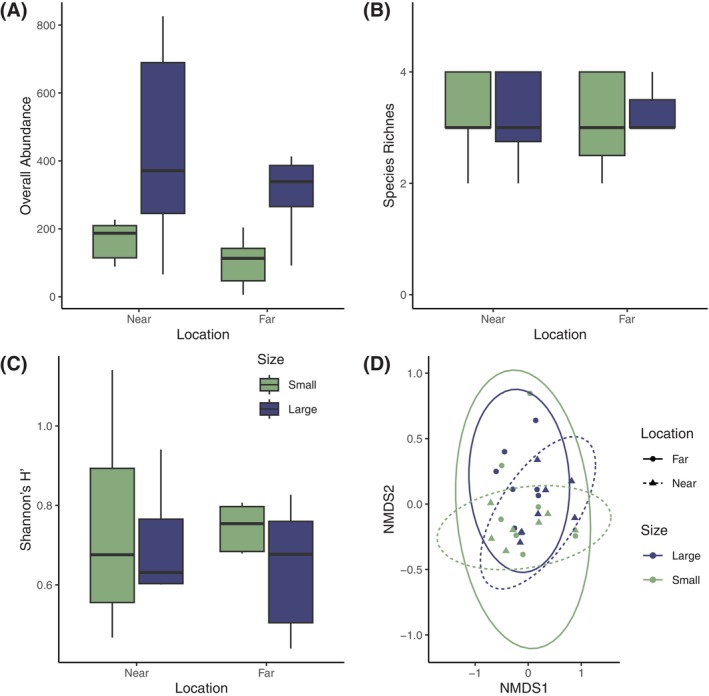
Sessile community settlement responses to habitat size and location. (A) shows the overall abundance of settled organisms per cage, (B) the number of species per cage, (C) the value for Shannon's H′ index of diversity, and (D) an nMDS plot based on Bray‐Curtis dissimilarities of the abundance of each of the four sessile organisms. Blue bars and points represent large cages (40 shells), and green bars and points represent small cages (20 shells). In panel (D), solid lines represent cages far from the restored reef, and dashed lines represent the cages near the restored reef.

Four sessile organisms colonized the shell: barnacles (*Balanus* spp.), oysters ( *Crassostrea virginica* ), hooked mussels ( *Ischadium recurvum* ), and serpulid worms (Serpulidae), which we were unable to identify to species in the field, but based on location were likely *Ficopomatus enigmaticus* or *Hydroides dianthus* (Bastida‐Zavala et al. 2017). Oysters were the most abundant (4849), followed by *Balanus* spp. (2576), *I. recurvum* (450), and Serpulidae (158). The overall number of individuals per cage was 74% higher in the large cages with 40 shells compared to small cages with 20 shells (Figure 2A, *t*(24) = −2.37, *p* = 0.026). Still, we did not detect a significant effect of location (*t*(24) = 1.19, *p* = 0.246) or an interaction between size and location (*t*(24) = 0.014, *p* = 0.989). The blocking term significantly affected overall abundance (*t*(25) = −3.071, *p* = 0.005). Additionally, we observed an effect of habitat size on community composition (Figure 2D, *p* = 0.005) but not an effect of location (*p* = 0.279) or block (*p* = 0.106).

While there was variation in overall abundance and community composition based on habitat size, there was no effect on diversity metrics such as species richness or Shannon's H' (Figure 2B,C). On average, all cages had approximately three species present, regardless of size (*t*(24) = −0.336, *p* = 0.740), location (*t*(24) = −0.395, *p* = 0.696), or block (*t*(24) = −0.034, *p* = 0.973). Similarly, average values for Shannon's H' ranged from 0.56 to 0.67, and we did not detect an effect of size (*t*(24) = 1.070, *p* = 0.295), location (*t*(24) = 0.260, *p* = 0.796), or block (*t*(24) = 0.308, *p* = 0.760) on Shannon's H'.

**FIGURE 3:**
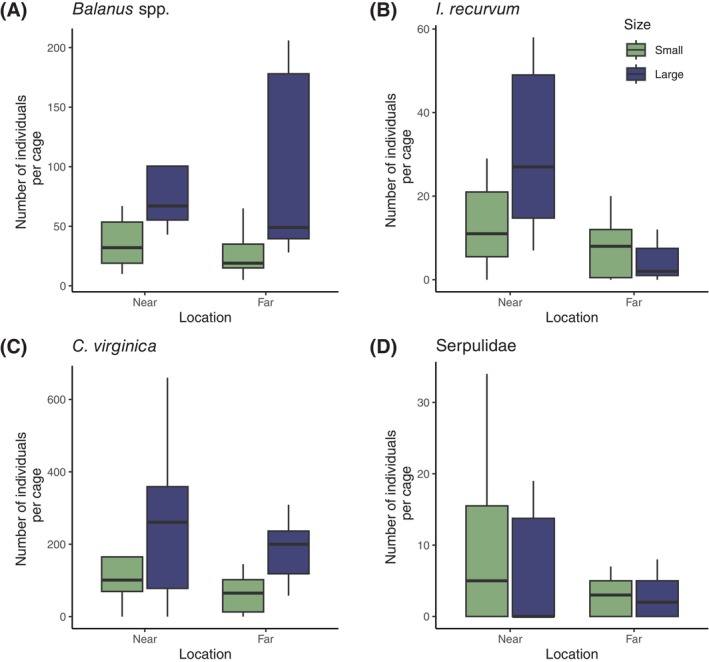
Abundance of four sessile organisms: *Balanus* spp. (A), *I. recurvum* (B), *C. virginica* (C), and Serpulidae (D) in response to habitat size and location. Blue bars represent large cages (40 shells), and green bars represent small cages (20 shells).

We observed species‐specific variation across both habitat size and location. The abundance of the two organisms largely depended on the cages' proximity to the restored reef. *I. recurvum* was 2.9 times more abundant in habitats near the restored oyster reef than those at a greater distance (Figure 3B, *z*(23) = 2.999, *p* = 0.0002). However, doubling habitat size did not significantly increase the abundance of *I. recurvum* (*z*(23) = 0.502, *p* = 0.616). Similarly, Serpulidae were more abundant in habitats near the restored reef, with cages near the restored reef having almost twice the number of worms as cages far from the reef (Figure 3D, *z*(22) = 3.078, *p* = 0.002). Increasing habitat size, though, had a negligible effect on Serpulidae numbers (*z*(22) = −0.0318, *p* = 0.750). Neither the abundances of *I. recurvum* nor Serpulidae showed an interaction between habitat location and size (*z*(23) = −1.197, *p* = 0.231, and *z*(22) = −0.075, *p* = 0.940, respectively).

The other two organisms, *Balanus* spp. and *C. virginica* , responded to habitat size. Cages with 40 shells had 3.8 times the barnacles as small cages with only 20 shells (Figure 3A, *z*(23) = −3.401, *p* = 0.0004). *C. virginica* were, on average, 1.4 times more abundant in large cages than in small cages (Figure 3C, *z*(22) = −2.297, *p* = 0.0216). Neither the number of *Balanus* spp. nor *C. virginica* varied by habitat location (*z*(23) = 0.373, *p* = 0.952 and *z*(22) = 1.591, *p* = 0.112, respectively), and neither *Balanus* spp. (*z*(23) = 0.875, *p* = 0.382) nor *C. virginica* (*z*(22) = 0.317, *p* = 0.751) had an interaction between size and location. Additionally, *Balanus* spp. had a significant blocking term (*z*(23) = −3.191, *p* = 0.0014).

## 4 Discussion

Organisms with complex life cycles, such as marine invertebrates with planktonic larvae, are often influenced by multiple factors when selecting a patch in a heterogeneous landscape. In this study, we tested the effects of habitat size and location. We found that both played a role in the distribution of sessile fauna on newly added shell near a restored oyster reef but that the specific response was organism‐dependent (Figure 3). The numbers of two organisms, *Balanus* spp. and *C. virginica*, were most affected by habitat size, while the numbers of Serpulidae and *I. recurvum* depended on habitat location. However, in our group of four sessile invertebrates, these patch characteristics had little to no effect on diversity (Figure 2).

Size affected the abundance of two of the sessile invertebrates. For one invertebrate, oysters, the increase in abundance as habitat size increased was approximately proportional, in line with the field of dreams hypothesis (Palmer et al. 1997), and as observed for oyster larvae in previous work (Kimbro et al. 2020). Barnacles, however, increased at a greater rate than the increases in habitat size. These organisms are known to be gregarious and are attracted to cues from conspecifics that likely drive these aggregations (Dreanno et al. 2006a) on top of the increase in abundance as habitat increases. Regarding the effect of location, hooked mussels and Serpulidae were more abundant near the restored reef (Figure 3B,D). This pattern suggests more of a “settlement shadow” type of effect where larvae are depleted as they move over existing habitat (Gaines et al. 1985) rather than something akin to propagule redirection where more isolated habitats gain more settlers because all arrivals to an area are allocated among existing habitat (Stier and Osenberg 2010). Interestingly, barnacles in this system did not exhibit a “settlement shadow” as they have in other studies, particularly on the western coast of the United States (Gaines et al. 1985).

The sessile community in our cages was similar to that found in other studies conducted in neighboring areas. For example, *Balanus* spp., Serpulidae, and *C. virginica* were found by Thomsen et al. 2007, but our study included hooked mussels rather than bryozoans. This study also showed low levels of variation among animals across sites at a larger spatial scale than ours. Yet, we saw a significant blocking term for two of our animals, suggesting high variation in larval supply due to specific aspects of the restored reef or hydrodynamics in our small study area.

Our study does not address temporal patterns in the assembly of these sessile communities, and our single collection at the end of the summer could mask transient patterns in settlement. Because some organisms colonize shell before others, later settling organisms might have fewer opportunities to settle as earlier colonizers occupy space and could be affected by the presence of conspecifics or heterospecifics. For example, *Balanus* spp. facilitates *C. virginica* settlement (Barnes et al. 2010). Interactions among species that have colonized the shell could enhance settlement patterns if organisms are attracted to organisms that settled according to a previous pattern or diminished if they settle in areas with more open space or avoid or compete with previously settled organisms. For example, the positive relationship between hooked mussels and oysters is positive and sigmoidal on artificial reef structure (Lipcius and Burke 2018), but studies of disturbance on benthic communities show that when disturbance is low, oysters can inhibit other sessile species due to space competition (Kimbro and Grosholz 2006). Therefore, the patterns from this study for each focal species might change in the absence of heterospecifics.

As is often the case with field studies, our data was quite noisy. The model of each species' abundance required a negative binomial distribution to account for heterogeneity in the counts, and some signal could be masked due to additional variation introduced over the summer. Attractants for settlers, such as chemical cues, can also be detectable in the lab but not in the field (Carroll et al. 2015; Pruett and Weissburg 2019). Hydrological factors, even on small spatial scales, can also affect settlement by oyster reef inhabitants (Fuchs et al. 2015), contributing to variation in settlement numbers among cages regardless of treatment.

While our study lasted longer than short‐term settlement studies (e.g., Eggleston et al. 1999), the study was still only 3 months long. Therefore, we did not capture the stable ecological community that would form over a longer time frame (Dillon et al. 2015; Rezek et al. 2017). However, settlement patterns such as those driven by the arrangement of habitat can produce long‐lasting variation (Hamman et al. 2018) and are reflected in equilibrium theories of biodiversity (MacArthur and Wilson 1967). Additionally, our experiment took place only during the summer months. While this matches the highest amount and diversity of colonizers in the Chesapeake Bay, we did not capture the colonization of some groups such as bryozoans and hydrozoans that primarily recruit in the winter and spring months (Jewett et al. 2022). Therefore, while it is likely that the observed patterns from our study contribute to variation across habitat patches based on size and location, further work incorporating the larger community is necessary to determine the long‐term trajectory of sessile invertebrate communities on oyster shell habitats.

Finally, our study only included two levels of habitat size and distance from the restored reef. Previous work has noted that there are often diminishing returns with habitat addition, indicating the relationship is nonlinear (Kimbro et al. 2020). Furthermore, marine larvae often incorporate a variety of cues, including physical, chemical, and biological, across multiple spatial scales in choosing their settlement habitat (Kingsford et al. 2002; Le Tourneux and Bourget 1988). Therefore, the results of habitat size and location at one spatial scale might differ from another. Future work should include a larger gradient of these responses to determine the shape of the relationships and the interactions between habitat size and location.

## Author Contributions

**Elizabeth A. Hamman:** conceptualization (lead), data curation (lead), formal analysis (lead), investigation (lead), methodology (lead), visualization (lead), writing – original draft (lead), writing – review and editing (lead).

## Conflicts of Interest

The author declares no conflicts of interest.

## Data Availability

The data and code used for analysis are currently available at https://figshare.com/s/6df1e4c4f9124849aaf8.
